# Limb body wall complex, amniotic band sequence, or new syndrome caused by mutation in *IQ Motif containing K* (*IQCK*)?

**DOI:** 10.1002/mgg3.153

**Published:** 2015-05-06

**Authors:** Paul Kruszka, Annette Uwineza, Leon Mutesa, Ariel F Martinez, Yu Abe, Elaine H Zackai, Rebecca Ganetzky, Brian Chung, Roger E Stevenson, Robert S Adelstein, Xuefei Ma, James C Mullikin, Sung-Kook Hong, Maximilian Muenke

**Affiliations:** 1 Medical Genetics Branch, National Human Genome Research Institute, National Institutes of HealthBethesda, Maryland; 2 Center for Medical Genetics, College of Medicine and Health Sciences, University of RwandaHuye, Rwanda; 3 Division of Human Genetics, The Children’s Hospital of Philadelphia, Clinical Genetics Center, Perelman School of Medicine of the University of PennsylvaniaPhiladelphia, Pennsylvania; 4 Department of Paediatrics and Adolescent Medicine, LKS Faculty of Medicine, The University of Hong KongPokfulam, Hong Kong; 5 Greenwood Genetic CenterGreenwood, South Carolina; 6 Laboratory of Molecular Cardiology, National Heart Lung and Blood Institute, National Institutes of HealthBethesda, Maryland; 7 Comparative Genomics Analysis Unit, National Human Genome Research Institute, National Institutes of HealthBethesda, Maryland

**Keywords:** Amniotic bands, ectopia cordis, limb anomalies, ventral midline defect

## Abstract

Limb body wall complex (LBWC) and amniotic band sequence (ABS) are multiple congenital anomaly conditions with craniofacial, limb, and ventral wall defects. LBWC and ABS are considered separate entities by some, and a continuum of severity of the same condition by others. The etiology of LBWC/ABS remains unknown and multiple hypotheses have been proposed. One individual with features of LBWC and his unaffected parents were whole exome sequenced and Sanger sequenced as confirmation of the mutation. Functional studies were conducted using morpholino knockdown studies followed by human mRNA rescue experiments. Using whole exome sequencing, a de novo heterozygous mutation was found in the gene *IQCK*: c.667C>G; p.Q223E and confirmed by Sanger sequencing in an individual with LBWC. Morpholino knockdown of *iqck* mRNA in the zebrafish showed ventral defects including failure of ventral fin to develop and cardiac edema. Human wild-type *IQCK* mRNA rescued the zebrafish phenotype, whereas human p.Q223E *IQCK* mRNA did not, but worsened the phenotype of the morpholino knockdown zebrafish. This study supports a genetic etiology for LBWC/ABS, or potentially a new syndrome.

## Introduction

Limb body wall complex (LBWC) is a usually fatal multiple congenital anomaly condition with craniofacial, limb, and ventral wall defects. The most accepted definition of LBWC has been meeting two of the following criteria: (1) thoraco-abdominoschisis or abdominoschisis; (2) limb defects; and, (3) craniofacial defects such as cleft lip/palate and encephalocele (Van Allen et al. 1987a,b); however, most clinicians consider a body wall defect as a minimum requirement of LBWC. Other anomalies associated with LBWC include complex heart defects, congenital scoliosis, spina bifida, and short umbilical cord. LBWC is a rare condition with a birth prevalence of 0.2–3.3/10,000 live births (Martínez-Frías 1997a; Luehr et al. 2002). The etiology remains unknown and recurrence rate is low.

Many of the anomalies of LBWC are found in amniotic band sequence (ABS) including craniofacial malformations, limb amputation defects, constriction rings, and neural tube defects (Lockwood et al. 1989; Bamforth 1992; Moerman et al. 1992; ten Donkelaar et al. 2002). Amniotic bands are formed from strings of ruptured amnion and are believed by some to entangle and constrict body parts causing amputation-like or constriction disruptions; however, ABS disruptions have been found in the absence of amniotic bands and not considered for the diagnosis of LBWC (Gazolla et al. 2014; Hartwig et al. 1989; Hunter et al. 2011; Luehr et al. 2002; Streeter 1930; Van Allen et al. 1987a,b). It remains unclear whether LBWC and ABS represent a single condition or two distinct disorders (Martínez-Frías 1997b; Halder 2010). For years, many different etiologies have been entertained for LBWC and the less severe phenotype, ABS (Streeter 1930; Torpin 1965; Van Allen et al. 1987a,b; Levy et al. 2007; Hunter et al. 2011). Hunter et al. (2011) has done a comprehensive review of these hypotheses.

Besides ABS, LBWC overlaps with a number of other conditions including pentalogy of Cantrell and Goltz–Gorlin syndrome (Focal Dermal Hypoplasia). Cantrell et al. (1958) described criteria in 1958 for combined congenital defects of the anterior abdominal wall, sternum, diaphragm, pericardium, and heart: (1) midline supra-umbilical abdominal defect, (2) defect of the lower sternum, (3) deficiency of the diaphragmatic pericardium, (4) deficiency of the anterior diaphragm, and (5) congenital intracardiac abnormality. Although not in the original description by Cantrell et al. a number of individuals with ectopia cordis and limb anomalies or facial clefting have been reported (Vanamo et al. 1991; Vazquez-Jimenez et al. 1998). Pivnick et al. (1998) described an infant with ectopia cordis, split right hand and left foot, bilateral cleft palate, and various other anomalies and acknowledged that this case had overlapping features of thoracoabdominal syndrome, pentalogy of Cantrell, and LBWC. In at least three reported cases of individuals with phenotypes of both Goltz–Gorlin syndrome and pentalogy of Cantrell, a mutation in *PORCN* has been found (Maas et al. 2009; Smigiel et al. 2011).

We can now confirm a genetic etiology, *IQ Motif Containing K* (*IQCK*), for a patient who fits some characteristics of ABS and LBWC or may represent a new syndrome. Mutations of *IQCK* have not been previously described in the medical literature.

## Patient Presentation and Methods

### Patient

The human studies were approved by the NHGRI institutional review board as part of the following US NIH study: 11-HG-0093 “Personalized Genomic Research” and was performed in compliance with US 45.CFR.46. All participants signed written informed consent for their participation.

**Figure 1:**
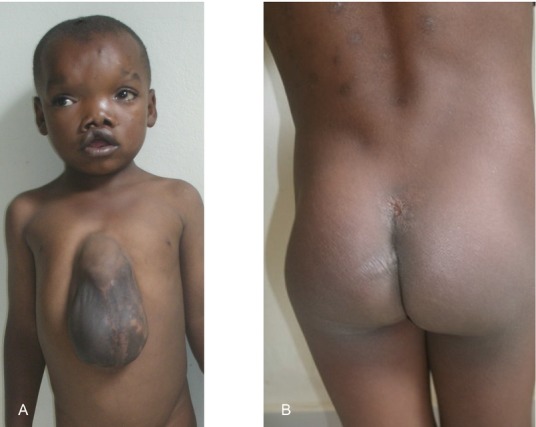
(A) Frontal view showing ventral midline defect; (B) postnatally acquired skin lesions.

Patient 1 is the fourth born of six children from healthy parents. There were no birth complications, and no noted amniotic bands. The patient possessed multiple congenital anomalies (Figs.4) including a 2 cm skin pedicle on his scalp, hypertelorism, depressed nasal bridge, broad nasal root, right cleft lip, large ventral wall defect, spina bifida occulta, and multiple limb anomalies. The ventral wall defect was skin, and not amnion covered. The limb anomalies consisted of right hand amputation-like defect of the 4th and 5th digits at the proximal phalanx (Figs.3B and C, 4A), cutaneous syndactyly of the 2nd and third digit of the left hand (Figs.3C and D, 4B), left congenital talipes equinovarus (Figs.3E, 4C), and bilateral split feet (Figs.3C, 4C and D). The patient had constriction rings on his left forearm and left great toe (Fig.3E and F). Multiple skin lesions were present on his back (Fig.1B), but these were acquired postnatally. An abdominal CT further clarified the anatomy of the ventral wall defect, which showed the right ventricle and part of the left ventricle outside the thoracic cavity and a diastasis of the abdominal musculature with colon protrusion. No diaphragmatic hernia or disruption was found. The sternum, scapula, ribs, liver, spleen, pancreas, adrenals, kidneys, and small bowel were normal. An echocardiogram of the heart showed an atrial septal defect (ASD) and a ventricular septal defect (VSD).

**Figure 2:**
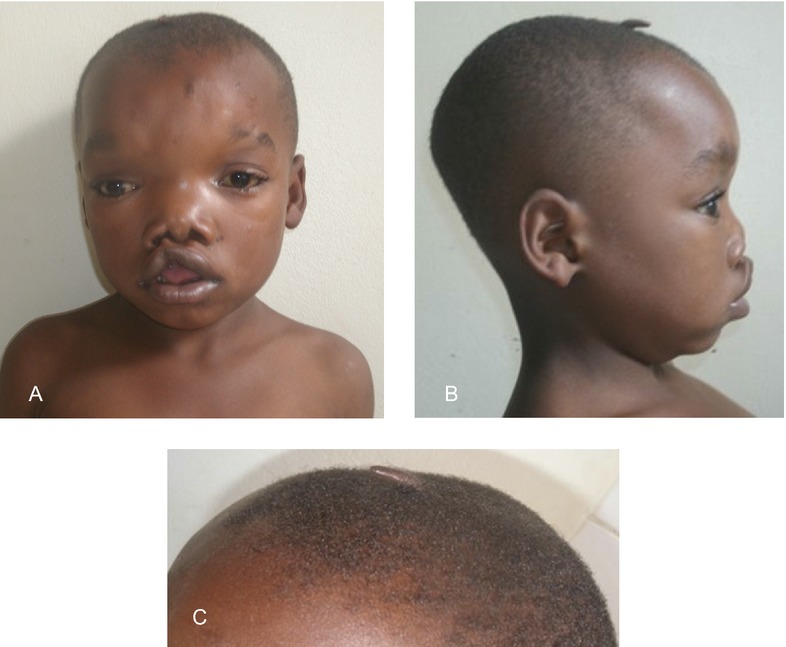
(A) Facial characteristics significant for hypertelorism, right cleft lip, short columella; (B) side profile showing scalp skin pedicle, depressed nasal bridge, malformed ear lobe and short columella; (C) scalp skin pedicle.

**Figure 3:**
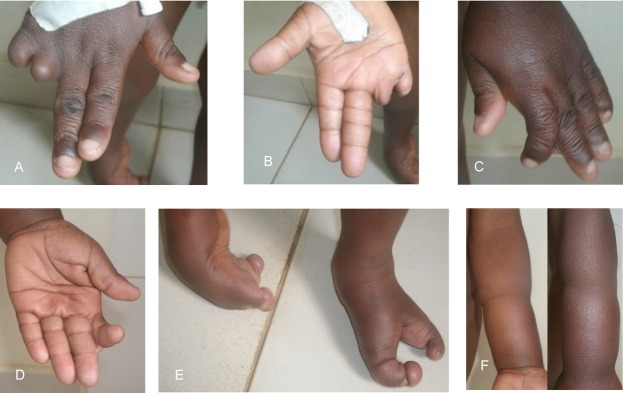
(A) Dorsal view of right hand showing terminal defect of 4th and 5th digits; (B) ventral right hand; (C) dorsal left hand shown cutaneous syndactyly of digits 2 and 3; (D) ventral left hand; (E) bilateral split feet, left congenital talipes equinovarus, constriction band left great toe; (D) circumferential constriction band mid-distance between elbow and wrist joints.

**Figure 4:**
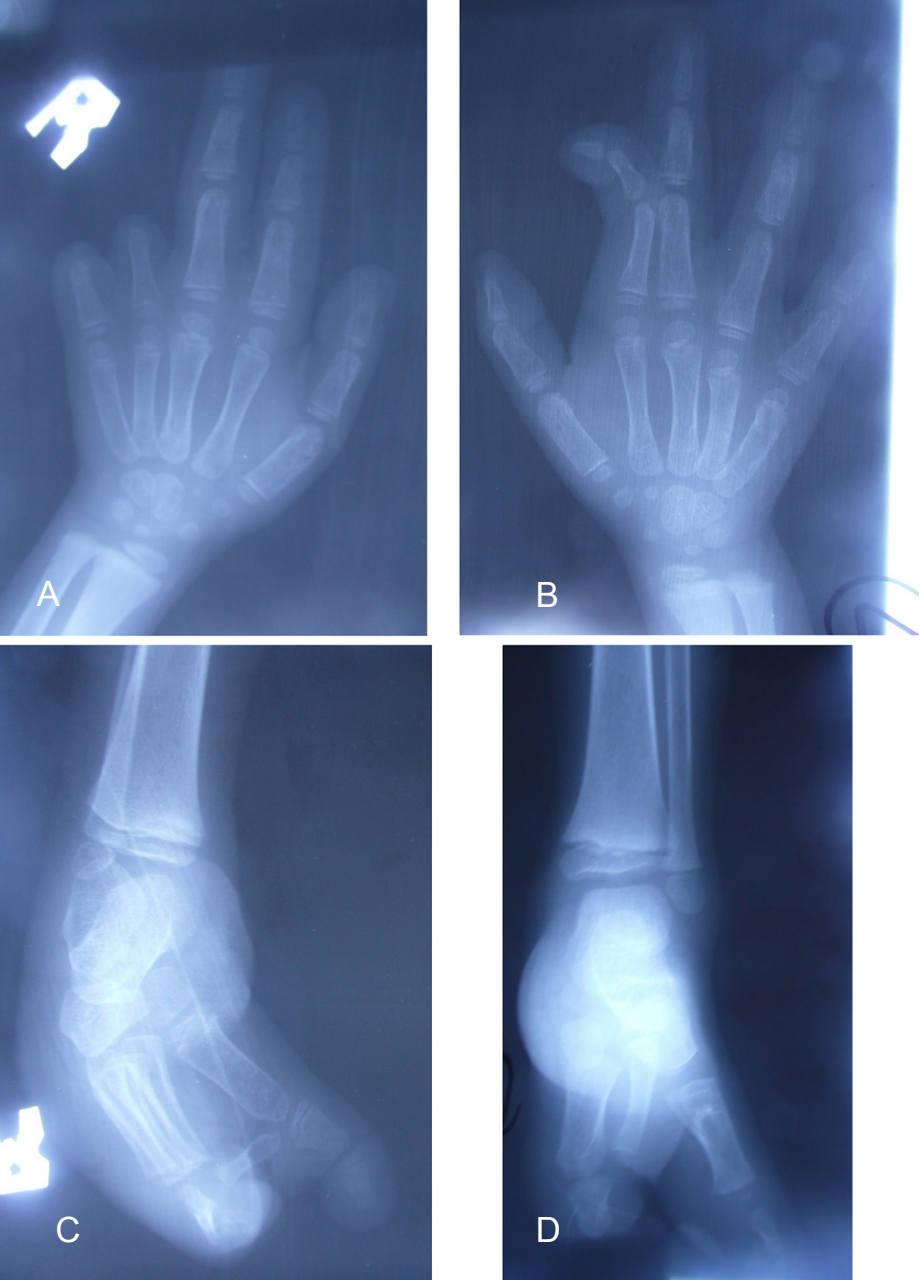
(A) X-ray of right hand demonstrating absence of middle and distal phalanges of digits 4 and 5; (B) x-ray of left hand shows cutaneous syndactyly of digits 2 and 3; (C) split right foot with missing metatarsal bones; (D) split left foot with malformed metacarpal bones and left congenital talipes equinovarus.

Patient 2 is an infant with LBWC complex identified and consented to our protocol for whole exome sequencing. This infant had many similar features to the previously described patient including a scalp skin pedicle, ectopia cordis, a large ASD and VSD requiring closure, and limb constriction bands. This infant also had a diaphragmatic hernia, aplasia cutis congenital of the left scalp, choanal atresia requiring surgery, bilateral iris colobomas, corneal clouding, and anterior lens subluxation. Additionally, 11 fetuses with LBWC and 17 cases of pentalogy of Cantrell were Sanger sequenced for evidence of an *IQCK* mutation.

### DNA extraction

Genomic DNA was isolated from whole blood using the QIAamp DNA Blood Maxi Kit (QIAGEN, Valencia, CA) following the manufacturer’s instructions. DNA samples were further prepared for next-generation sequencing by phenol/chloroform extraction.

### Next-generation sequencing

Exome sequencing, assembly, genotyping, and annotation were carried out on the family trio with LBWC and one singleton with LBWC by the National Intramural Sequencing Center (NISC) using genomic enrichment (Johnston et al. 2010; Teer et al. 2010). Capture utilized the NimbleGen SeqCap EZ Version 3.0+ UTR (Roche NimbleGen, Madison, WI). Captured regions totaled approximately 96 Mb. Flow cell preparation and 125-bp paired end read sequencing were performed as per the HiSeq2000 Sequencer protocol (Illumina, San Diego, CA). The percentage of the Consensus Coding Sequence exome with most probable genotype quality scores of 10 exceeded 85%. DNA variant list manipulation was performed using VarSifter (Teer et al. 2012).

### Sanger sequence analysis

*IQCK* sequence verification was performed using standard methods (Sanger et al. 1977). Sequencing was performed with v3.1 BigDye Terminator Cycle Sequencing Kit (Life Technologies, Grand Island, NY) in the ABI 3730xl Sequencer (Life Technologies) according to the manufacturer’s protocol. Sequence data were aligned to the published reference genomic sequence for *IQCK* (GenBank accession number NC_000016.10) using Sequencher 5.0.1 (Gene Codes Corp., Ann Arbor, MI).

### Zebrafish assay

10 ng of zebrafish *iqck* ATG morpholino (MO); 5′-AGTCAGACTCGCTCATGCTGGTCTC-3′ and standard control MO were synthesized by Gene Tools (Philomath, OR). MOs were injected into the one-cell stage of fertilized embryos and the phenotype was observed at 48 h. For the MO rescue, experiments were conducted with 150 pg of human *IQCK* mRNA, including wild type, p.Q223E, p.Q223H, and p.Q222S variants RNAs, mixed with 10 ng of *iqck* ATG MO. All zebrafish experiments were performed at least 10 times.

## Results

**Figure 5:**
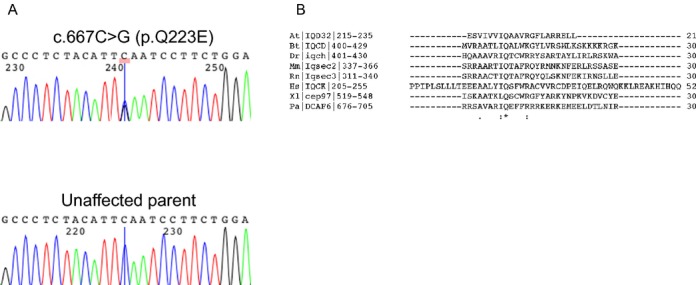
(A) Sanger sequencing chromatograms show *IQCK* wild-type sequence found in parents of patient 1 and the heterozygous missense mutation found in patient 1; (B) Protein sequence alignment of human IQCK with several IQ motif-containing proteins show absolute conservation of the glutamine (Q) residue across species (denoted by an asterisk). This is residue found mutated in our patient (p.Q223E). Pa: *Pongo abelii* (Sumatran orangutan); At: *Arabidopsis thaliana* (Mouse-ear cress); Dr: *Danio rerio* (Zebrafish); Bt: *Bos taurus* (Bovine); Xl: *Xenopus laevis* (African clawed frog); Hs: Homo sapiens (Human); Rn: Rattus norvegicus (Rat); Mm: Mus musculus (Mouse). The gene symbol is indicated between vertical lines, with the numbers indicating the amino acid positions in the reference isoform of the protein (isoform 1). Sequence alignment was performed using the CrustalW2 tool from EMBL-EBI (http://www.ebi.ac.uk/Tools/msa/clustalw2).

Using whole exome sequencing, a de novo heterozygous mutation was found in the gene *IQCK*: c.667C>G; p.Q223E in Patient 1. This occurs in a conserved amino acid in the IQ domain of *IQCK*. This variant was verified by Sanger sequencing (Fig.5A) in the proband but was absent in both parents. Multiple prediction models were used to evaluate the potential pathogenicity of the variant, including a Combined Annotation-Dependent Depletion (CADD) score of 39.0, a Grantham score of 29, a genomic evolutionary rate profiling (GERP) score of 5.6, and a PolyPhen-2 (Polymorphism Phenotyping v2) prediction of “probably damaging.” This mutation was not found in multiple databases including 1000 Genomes, the Exome Aggregation Consortium (ExAC), and the NHLBI Exome Sequencing Project (ESP).

Sequence alignment of human IQCK with IQ motif-containing proteins from vertebrate and invertebrate species, and a plant species (*Arabidopsis thaliana*), shows that the glutamine residue is the only amino acid strictly conserved across species (Fig.5B).

**Figure 6:**
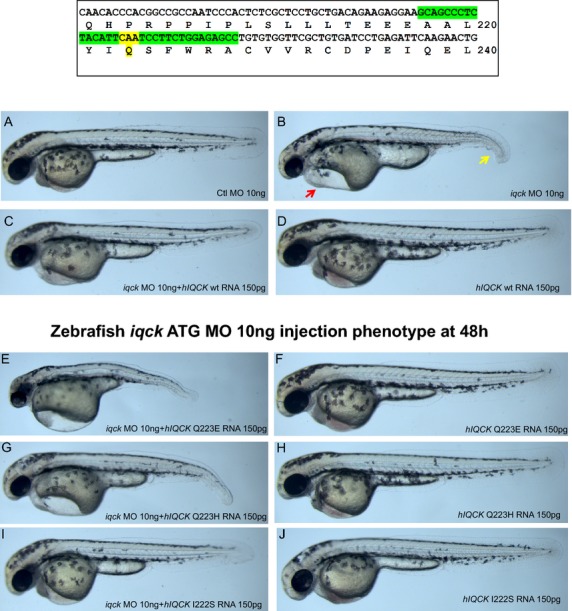
Box shows location of missense mutation in *IQCK;* (A) control morpholino phenotype at 48 h in zebrafish is normal; (B) *iqck* morpholino at 48 h shows cardiac edema (red arrow) and ventral fin hypoplasia (yellow arrow); (C) coinjection of *iqck* morpholino and *IQCK (hIQCK)* mRNA shows rescued phenotype; (D) control human *IQCK* injection results in normal phenotype; (E) coinjection of *iqck* morpholino and human Q223E human *IQCK* mRNA resulting in worsening of phenotype than *iqck* morpholino alone shown in Figure6B; (F) human Q223E *IQCK* mRNA injection shows normal phenotype; (G) coinjection of *iqck* morpholino and human Q223H human *IQCK* mRNA resulting failure to rescue phenotype; (H) human Q223H *IQCK* mRNA injection shows normal phenotype; (I) coinjection of *iqck* morpholino and human Q222S human *IQCK* mRNA resulting in a rescued phenotype; (J) human Q222S *IQCK* mRNA injection shows normal phenotype.

Functional studies using MO knockdown showed a significant phenotype in the zebrafish embryo at 48 h past fertilization (hpf). While the control MO showed a normal phenotype (Fig.6A), the *iqck* morpholino showed cardiac edema and poor development of the ventral fin (Fig.6B). Coinjection of human *IQCK* wild-type mRNA rescued the *iqck* MO phenotype (Fig.5C) with reduction in heart edema and normal growth of ventral part of fin, (Fig.6C), whereas human p.Q223E mRNA worsened this phenotype (Fig.6E) with markedly decreased tail development and increased cardiac edema. However, when the human p.Q223E mRNA was administered without the *iqck* MO, the zebrafish phenotype was unaffected (Fig.6F). When rescue of the *iqck* MO was attempted using histidine (H) versus glutamic acid (E) with p.Q223H human mRNA, the phenotype remained unchanged (not rescued) (Fig.6G). Coinjection of p.I222S human mRNA (also a conserved amino acid in the IQ domain) only partially rescued the MO phenotype (Fig.6I). These results suggest that the conserved glutamine in position 223 is essential and that the p.Q223E mutation is pathogenic.

A second case of LBWC (singleton) was evaluated by whole exome sequencing, but no mutations were found in *IQCK* or *PORCN*. Additionally, 11 fetuses with LBWC and 17 cases of pentalogy of Cantrell were Sanger sequenced for the *IQCK* gene, but no mutations were found.

## Discussion

Here, we present a carefully phenotyped case (Patient 1) found to have three elements considered by Van Allen et al. (1987a,b) to fit the definition of LBWC, including cleft lip, thoraco-abdominoschisis, and limb anomalies. However, given that LBWC is almost universally lethal, especially when a ventral wall defect is present, the described case would not be considered LBWC by some experienced clinicians (Gazolla et al. 2014). This patient has elements of both LBWC and ABS, which is consistent with the overlap between these two syndromes; however, the medical literature has not determined whether these two conditions are separate nor has the causation of these two conditions been found.

Amniotic bands are plausible as an etiology given the left forearm and great toe constriction rings, and the 4th and 5th digit amputation-like findings in the right hand. However, amniotic band disruption does not explain the ventral wall defect, and atrial and ventricular septal defects found in our patient. LBWC is a more severe condition than ABS as it affects more organ systems, but they could be the same condition displaying variable expressivity. Levy et al. (2007) presented a mother with one child with LBWC and a subsequent infant with ABS, which supports a connection between these two conditions, whether genetic, environmental, or combination of both.

There are three proposed theories on the cause of ABS and/or LBWC. The first is the extrinsic theory proposed by Torpin, which considers early amniotic sac rupture followed by the formation of amniotic bands, which entrap and disrupt limb formation leading to amputations and constriction bands (Torpin 1965). This extrinsic model does not explain the internal organ (i.e., heart and kidneys) malformations often found with LBWC. The second proposed mechanism is vascular disruption causing the internal malformations seen in LBWC and the persistence of the extraembryonic coelom as the origin of amniotic bands (Van Allen et al. 1987a,b). The third theory proposed by Streeter (1930) focuses on an abnormality in the germinal disk that results in final structural malformations. Our case, which has elements of both ABS and LBWC, supports a genetic etiology as a cause ABS and LBWC. Additionally there are two cases of familial ABS in the literature (Lockwood et al., 1988) and twin studies support a genetic etiology for the LBWC/ABS spectrum (Lubinsky et al. 1983; Luehr et al. 2002).

Another interesting consideration is a human homologue to the mouse disorganization (Ds) mutation (Robin and Nadeau 2001). A number of reports in the literature with skin pedicles and ABS findings propose a disorganization hypothesis. Skin pedicles are a rare finding, and previous described cases with skin pedicles resemble the two patients presented with LBWC in this report (Fig.2B and C) in a number of ways (Isidor et al. 2009; Temtamy et al. 2010). Isidor et al. presented four patients with skin pedicles and severe limb anomalies that were originally diagnosed as ABS, and proposed that these individuals resemble the disorganization phenotype in the mouse. Donnai and Winter first suggested that patients with ABS resembled the mouse with the yet to be found disorganization (Ds) mutation, and more reports as with features of ABS have suggested a human homologue to Ds, including patients with skin pedicles and limb anomalies (Donnai and Winter 1989; Lowry and Yong 1991; Nakamura and Nanjyo 1992; Robin et al. 2005). In one case, a ventral wall defect was reported (Donnai and Winter 1989). The Ds phenotype described by Hummel consisted of mice with a heterozygous mutation, homozygous lethal, that had many similarities to the ABS phenotype and to our two cases including neural tube defects, hamartomas represented by papillomas protruding from the body, limb anomalies, ventral wall defects, and facial clefting (Hummel 1959). The skin pedicle (Fig.2B and C) in our case was not biopsied; however, ten Donkelaar et al. (2002) did biopsy a similar scalp lesion in a boy who also had amputation-like hand defects and the biopsy showed evidence of a rudimentary meningocele. The multiple congenital anomalies presented in addition to the skin pedicle makes the phenotype presented in this study unique and possibly a new syndrome.

As noted above, there have been a number of hypotheses for the cause of ABS and LBWC, and based on our data, ABS and/or LBWC may be caused by a genetic mutation. Here, we report the gene, *IQCK*, to be associated with a ABS/LBWC phenotype. Whole exome sequencing identified a de novo coding variant in this gene that was confirmed with Sanger sequencing. This variation introduces a nonsynonymous change in the IQ motif of the protein. The morpholino knockdown of the zebrafish shows cardiac edema, and failure of ventral fin to develop. The wild-type human *IQCK* mRNA rescues the morpholino knockdown zebrafish; however, the p.Q223E human IQCK mRNA worsens the phenotype of the morpholino knockdown zebrafish.

Although we have shown that the p.Q223E mutation is functionally important, the molecular function or pathway(s) of *IQCK* remain unknown. The p.Q223E mutation is located in a very conserved amino acid of the IQ domain (Fig.5B). The IQ domain is approximately 25 amino acids in length and is widely distributed in nature. The motif conforms to the more general consensus sequence [I,L,V]Qxxx[R,K]xxxx[R,K], which forms an amphiphilic seven-turn *α*-helix capable of binding calmodulin in a Ca^2+^-independent manner (Bähler and Rhoads 2002). Calmodulin mediates the effects of the major cellular second signal Ca^2+^ and can stimulate changes in the actin cytoskeleton mediated by proteins such as myosin. Nearly all myosin proteins possess between one and seven IQ domains, most found in multiple tandem repeats separated by 9–16 amino acid residues. Proteins that contain at least one IQ domain include myosins, voltage-gated channels, neuronal proteins (i.e., neuromodulin, PEP-19), phosphatases, sperm surface proteins, Ras exchange proteins, spindle-associated proteins, at least one RasGAP-like protein and several plant-specific proteins (Bähler and Rhoads 2002). In the Pfam protein families’ database, there are over 900 entries with IQ domains (Finn et al. 2014).

Although further research is needed to define IQCK function and its relationship with other proteins, a large number of recent literature reports support an active role of IQ motif-containing proteins in cell polarization and migration (Johnson and Henderson 2012; Choi et al. 2013; Jacquemet and Humphries 2013; Banon-Rodriguez et al. 2014; Foroutannejad et al. 2014; Naylor and Morgan 2014) and even in ciliary function (Cyr et al. 2002; Otto et al. 2005; Spektor et al. 2007), which introduces the possibility that LBWC could be a new ciliopathy.

Testing one other case with a similar LBWC phenotype and 11 additional cases with LBWC did not find a mutation in *IQCK*. Only a singleton was tested with whole exome sequencing, making gene discovery difficult. We hypothesize that like many syndromes, LBWC will be genetically heterogeneous. Seventeen samples of patients with pentalogy of Cantrell failed to show a mutation in IQCK, which may support pentalogy of Cantrell being a different condition than LBWC. Further research including next-generation sequencing of trios (affected proband and parents) and tissue-specific sequencing evaluating for mosaicism will be needed to further characterize this heterogeneous condition.

In summary, this study begins to answer the question of a genetic etiology of ABS and LBWC, supported by genetic testing and functional studies in zebrafish. The pathogenesis of LBWC and ABS is most likely heterogeneous based on the discussion above, and our case with an *IQCK* mutation may represent a new syndrome, with elements of LBWC but less severe and survivable. More cases with a similar phenotype will be needed to confirm *IQCK* as a causative gene and we suspect that more genes, possibly involved in the same cellular pathway, will be found. Further investigation of *IQCK* will be required to elucidate its molecular function and corroborate its implication in the etiology of LBWC.

## Conflict of Interest

None declared.

## References

[b1] Bähler M, Rhoads A (2002). Calmodulin signaling via the IQ motif. FEBS Lett.

[b2] Bamforth JS (1992). Amniotic band sequence: Streeter’s hypothesis reexamined. Am. J. Med. Genet.

[b3] Banon-Rodriguez I, Galvez-Santisteban M, Vergarajauregui S, Bosch M, Borreguero-Pascual A, Martin-Belmonte F (2014). EGFR controls IQGAP basolateral membrane localization and mitotic spindle orientation during epithelial morphogenesis. EMBO J.

[b4] Cantrell JR, Haller JA, Ravitch MM (1958). A syndrome of congenital defects involving the abdominal wall, sternum, diaphragm, pericardium, and heart. Surg. Gynecol. Obstet.

[b5] Choi S, Thapa N, Hedman AC, Li Z, Sacks DB, Anderson RA (2013). IQGAP1 is a novel phosphatidylinositol 4,5 bisphosphate effector in regulation of directional cell migration. EMBO J.

[b6] Cyr JL, Dumont RA, Gillespie PG (2002). Myosin-1c interacts with hair-cell receptors through its calmodulin-binding IQ domains. J. Neurosci.

[b7] ten Donkelaar HJ, Hamel BC, Hartman E, van Lier JA, Wesseling P (2002). Intestinal mucosa on top of a rudimentary occipital meningocele in amniotic rupture sequence: disorganization-like syndrome, homeotic transformation, abnormal surface encounter or endoectodermal adhesion?. Clin. Dysmorphol.

[b8] Donnai D, Winter RM (1989). Disorganisation: a model for ‘early amnion rupture’?. J. Med. Genet.

[b9] Finn RD, Bateman A, Clements J, Coggill P, Eberhardt RY, Eddy SR (2014). Pfam: the protein families database. Nucleic Acids Res.

[b10] Foroutannejad S, Rohner N, Reimer M, Kwon G, Schober JM (2014). A novel role for IQGAP1 protein in cell motility through cell retraction. Biochem. Biophys. Res. Commun.

[b11] Gazolla AC, da Cunha AC, Telles JA, Betat Rda S, Romano MA, Marshall I (2014). Limb-body wall defect: experience of a reference service of fetal medicine from Southern Brazil. Birth Defects Res. A Clin. Mol. Teratol.

[b12] Halder A (2010). Amniotic band syndrome and/or limb body wall complex: split or lump. Appl. Clin. Genet.

[b13] Hartwig NG, Vermeij-Keers C, De Vries HE, Kagie M, Kragt H (1989). Limb body wall malformation complex: an embryologic etiology?. Hum. Pathol.

[b14] Hummel KP (1959). Developmental anomalies in mice resulting from action of the gene Disorganization, a semi-dominant lethal. Pediatrics.

[b15] Hunter AG, Seaver LH, Stevenson RE (2011). Limb-body wall defect. Is there a defensible hypothesis and can it explain all the associated anomalies?. Am. J. Med. Genet. A.

[b16] Isidor B, Baujat G, Le Caignec C, Pichon O, Martin-Coignard D, Toutain A (2009). Congenital skin pedicles with or without amniotic band sequence: Extending the human phenotype resembling mouse disorganization. Am. J. Med. Genet. A.

[b17] Jacquemet G, Humphries MJ (2013). IQGAP1 is a key node within the small GTPase network. Small GTPases.

[b18] Johnson MA, Henderson BR (2012). The scaffolding protein IQGAP1 co-localizes with actin at the cytoplasmic face of the nuclear envelope: implications for cytoskeletal regulation. Bioarchitecture.

[b19] Johnston JJ, Teer JK, Cherukuri PF, Hansen NF, Loftus SK, Chong K, Mullikin JC, Biesecker LG, NIH Intramural Sequencing Center (NISC) (2010). Massively parallel sequencing of exons on the X chromosome identifies RBM10 as the gene that causes a syndromic form of cleft palate. Am. J. Hum. Genet.

[b21] Levy R, Lacombe D, Rougier Y, Camus E (2007). Limb body wall complex and amniotic band sequence in sibs. Am. J. Med. Genet. A.

[b50] Lockwood C, Ghidini A, Romero R (1988). Amniotic band syndrome in monozygotic twins: prenatal diagnosis and pathogenesis. Obstet Gynecol.

[b22] Lockwood C, Ghidini A, Romero R, Hobbins JC (1989). Amniotic band syndrome: reevaluation of its pathogenesis. Am. J. Obstet. Gynecol.

[b23] Lowry RB, Yong SL (1991). Cleft lip and palate, sensorineural deafness, and sacral lipoma in two brothers: a possible example of the disorganisation mutant. J. Med. Genet.

[b24] Lubinsky M, Sujansky E, Sanger W, Salyards P, Severn C (1983). Familial amniotic bands. Am. J. Med. Genet.

[b25] Luehr B, Lipsett J, Quinlivan JA (2002). Limb-body wall complex: a case series. J. Matern Fetal Neonatal. Med.

[b26] Maas SM, Lombardi MP, van Essen AJ, Wakeling EL, Castle B, Temple IK (2009). Phenotype and genotype in 17 patients with Goltz-Gorlin syndrome. J. Med. Genet.

[b27] Martínez-Frías ML (1997a). Clinical and epidemiological characteristics of infants with body wall complex with and without limb deficiency. Am. J. Med. Genet.

[b28] Martínez-Frías ML (1997b). Epidemiological characteristics of amniotic band sequence (ABS) and body wall complex (BWC): are they two different entities?. Am. J. Med. Genet.

[b29] Moerman P, Fryns JP, Vandenberghe K, Lauweryns JM (1992). Constrictive amniotic bands, amniotic adhesions, and limb-body wall complex: discrete disruption sequences with pathogenetic overlap. Am. J. Med. Genet.

[b30] Nakamura K, Nanjyo B (1992). Congenital skin tube pedicle associated with the constriction band syndrome. Plast. Reconstr. Surg.

[b31] Naylor SG, Morgan DO (2014). Cdk1-dependent phosphorylation of Iqg1 governs actomyosin ring assembly prior to cytokinesis. J. Cell Sci.

[b32] Otto EA, Loeys B, Khanna H, Hellemans J, Sudbrak R, Fan S (2005). Nephrocystin-5, a ciliary IQ domain protein, is mutated in Senior-Loken syndrome and interacts with RPGR and calmodulin. Nat. Genet.

[b33] Pivnick EK, Kaufman RA, Velagaleti GV, Gunther WM, Abramovici D (1998). Infant with midline thoracoabdominal schisis and limb defects. Teratology.

[b34] Robin NH, Nadeau JH (2001). Disorganization in mice and humans. Am. J. Med. Genet.

[b35] Robin NH, Franklin J, Prucka S, Ryan AB, Grant JH (2005). Clefting, amniotic bands, and polydactyly: a distinct phenotype that supports an intrinsic mechanism for amniotic band sequence. Am. J. Med. Genet. A.

[b36] Sanger F, Nicklen S, Coulson AR (1977). DNA sequencing with chain-terminating inhibitors. Proc Natl Acad Sci U S A.

[b37] Smigiel R, Jakubiak A, Lombardi MP, Jaworski W, Slezak R, Patkowski D (2011). Co-occurrence of severe Goltz-Gorlin syndrome and pentalogy of Cantrell - Case report and review of the literature. Am. J. Med. Genet. A.

[b38] Spektor A, Tsang WY, Khoo D, Dynlacht BD (2007). Cep97 and CP110 suppress a cilia assembly program. Cell.

[b39] Streeter GL (1930). Focal deficiencies in fetal tissues and their relation to intrauterine amputations. Contrib Embryol.

[b40] Teer JK, Bonnycastle LL, Chines PS, Hansen NF, Aoyama N, Swift AJ (2010). Systematic comparison of three genomic enrichment methods for massively parallel DNA sequencing. Genome Res.

[b41] Teer JK, Green ED, Mullikin JC, Biesecker LG (2012). VarSifter: visualizing and analyzing exome-scale sequence variation data on a desktop computer. Bioinformatics.

[b42] Temtamy SA, Aglan MS, Ashour AM, El-Badry TH (2010). Limb malformations with associated congenital constriction rings in two unrelated Egyptian males, one with a disorganization-like spectrum and the other with a probable distinct type of septo-optic dysplasia. Clin. Dysmorphol.

[b43] Torpin R (1965). Amniochorionic mesoblastic fibrous strings and amnionic bands: associated constricting fetal malformations or fetal death. Am. J. Obstet. Gynecol.

[b44] Van Allen MI, Curry C, Gallagher L (1987a). Limb body wall complex: I. Pathogenesis. Am J Med Genet.

[b45] Van Allen MI, Curry C, Walden CE, Gallagher L, Patten RM (1987b). Limb-body wall complex: II. Limb and spine defects. Am. J. Med. Genet.

[b46] Vanamo K, Sairanen H, Louhimo I (1991). The spectrum of Cantrell’s syndrome. Pediatr. Surg. Int.

[b47] Vazquez-Jimenez JF, Muehler EG, Daebritz S, Keutel J, Nishigaki K, Huegel W (1998). Cantrell’s syndrome: a challenge to the surgeon. Ann. Thorac. Surg.

